# Christmas in the Hospitals

**Published:** 1917-01-06

**Authors:** 


### CHRISTMAS IN THE HOSPITALS.

### St. Bartholomew's Hospital.

There is no doubt that the war has in many ways altered the Christmas festivities at this hospital. Nevertheless, though altered, the proceedings were in no way inferior either in entertainment or decoration to those of pre-war days. The chief difference to be noticed was that whereas in normal times a very large part of the entertainment in the wards was provided by students who formed various "troupes," nowadays one has to rely upon outside talent for the most part, for, alas! the number of students is considerably reduced.

### An Imposing Father Christmas.

Father Christmas was in evidence, as usual, this year, and not for many a year has so fine a representative appeared; the impersonator is very tall himself, and with the flowing wig and straggling beard he appeared to be at least six feet four in height—an imposing and, to many of the children, an awe-inspiring figure. He it was who in the morning went from ward to ward and distributed from each Christmas-tree the presents to old and young. Among the children this is probably the most exciting and tremendous episode in the whole day of excitement and tremendous episodes of which Christmas consists for those of the very poor who have never experienced anything to compare with it. Each ward had its own Christmas-tree, and these were variously decorated and lighted. Some of the larger ones stood about nine feet from the ground, and were elaborately lighted by an ingenious arrangement of electric bulbs.

After Father Christmas came the mid-day dinner. Each ward had its turkey and plum-pudding, the latter in most cases aflame with fire in the approved old-fashioned style. Of course, there were many unfortunate patients (more especially in the medical wards) whose condition prohibited the season's fare. It was really pathetic in one instance to see a patient with a gastric ulcer look longingly at the food he might not touch, and one was inclined to wonder whether his desire might not produce a psychic flow of gastric juice of more bad influence than the sausages and turkey! Before the war a house surgeon or physician was wont to preside in the ward and carve the bird in approved style; during the last two years some of the senior students have taken their places, but this year in many wards it fell to the lot of the sister to handle the job; thus as one goes another fills the place, and most excellently too.

### A Feast of Concerts.

At three o'clock the performances in the wards began. There were nine concert troupes on the list of performers, but of these only eight were able to be present, and each of these did on the average seven "turns," of about twenty-five minutes each. In all, therefore, about fifty-six performances took place—no bad record for war-time.

Of the various troupes only one was recruited from the hospital, and this styled itself "The Dry Dressings." It comprised several of the junior staff and one or two senior students. However, what the hospital lacked in quantity it certainly gained in quality, for this was one of the best troupes which the hospital has produced for a long time; both its singing and dancing were excellent. Of the visitors who kindly provided amusement it would be difficult to pick out any one as better than the others, for the "turns" which they provided were of such variety that one cannot with justice compare them. Of the troupes that pleased us best, however, we may mention "The Rowland Ramblers." Who the ladies were, however, who provided this remarkable entertainment we do not know. Their "coster singing" was above reproach, and their dancing delightful in the extreme. The troupe provided by Mr. Seymour Dickers also deserves special mention, Miss Florence Jennings rendering her songs with a beautifully clear and dramatic voice. "Charlie's Aunt," for such we must call the gallant officer now dressed in the clothes of a very respectable charwoman, gave his songs in a manner worthy of a first-rate comedian, as indeed he is, although we understand with surprise that he is not in that profession.

Space will not permit us to mention all the troupes respectively, the "Pierrots" and those others, all of whom were in such good form. Moreover, be it confessed that with so much to see a great deal was left unseen.

About 6.30 most of the entertainments were brought to a close, save only for the Christmas dinner of the junior staff, which was much like every other Christmas dinner of a junior staff—merry and hearty.

### An Improvement—and Economy?

The wards were throughout decorated with much taste, the paper streamers and rosettes being in their usual evidence. Perhaps the chief change we noticed this year was the slight diminution of paper decorations on the ceiling in favour of Chinese lanterns; whether this was because the latter decoration is somewhat cheaper we do not know. In any case the effect struck us as an improvement—cheaper or not.

By 11.30 p.m. the usual darkness and sleep had supervened upon the gaieties of the day and—but patience a moment. Two babies were born in the hospital on this auspicious day. They alone will not carry memories into the future. Good luck to them!

### Manchester Royal Infirmary.

The Christmas festivities at the Manchester Royal Infirmary commenced, as usual, on Christmas Eve by the singing of carols by the choir of Manchester Cathedral throughout the various wards, which were tastefully decorated and presented a picture of real Christmastide. Christmas Day commenced by the celebration of Holy Communion in the infirmary chapel at 6.15 a.m. and 8.30 a.m., and Matins at 9.45 a.m. These services were well attended by the staff and patients, both military and civilian.

The great item of interest in the morning was the Christmas-tree in the out-patient department. At 10 o'clock close on 400 poor children who had previously received invitations assembled in the out-patients' hall to take part in the distribution of the toys adorning the Christmas-tree and of the fruit which had been provided for them. Many of the wounded soldiers in the hospital attended, and caused a considerable amount of fun by their inimitable humour. The scene was a remarkable one, and we hope to publish a photograph of it in an early issue. The tree formed a fine centre-piece, with a background of tables literally groaning under the weight of fruit and cakes, and the happy faces of the children were ample repayment to those who had contributed in making their treat a success; the whole proceeding passed off most happily. In the afternoon the resident medical officers, together with various

CHRISTMAS IN THE HOSPITALS—(*continued*).

friends, entertained the patients in their different wards, a piano being provided in each ward. After an exceptionally nice tea, games and music were indulged in until 8.30 P.M. Throughout the whole of the hospital the endeavours of those who had contrived to bring peace and good will were deeply appreciated, and there was an unmistakable feeling that this Christmas of 1916 would prove indeed a memorable one, as the precursor of a real peace in the near future.

### Leeds General Infirmary.

ALTHOUGH the outward and visible sign of Christmas, in the way of decoration, was considerably less than on previous occasions in the wards of the General Infirmary at Leeds, much was done to make the patients forget their pain and lessen the sadness at being parted from friends. The usual Christmas fare—turkeys, beef, Christmas pudding, cakes, etc.—was provided.

The resident medical staff and nurses entered fully into the spirit of the occasion, the former hiding their identity in character costumes.

The wounded soldiers were much cheered by the receipt of a telegram from Their Majesties the King and Queen.

On Christmas Eve and Christmas evening the nurses and maids promenaded the wards, singing carols and hymns, in which many patients joined.

INJURED MUNITION-MAKERS.

One ward, occupied entirely by women injured in a recent serious accident in a local engineering shop, received special consideration by other and more fortunate employees at the same works. Numerous costly presents were given to each, and in the case of women with families a present for each child was provided. The managing director of the works was present during dinner and carved, and arranged entertainments for the afternoon and evening. The patients appreciated the kind thoughtfulness which prompted the care bestowed upon them.

On the two subsequent evenings, in the out-patients' waiting hall (now occupied as a ward for eighty-seven wounded soldiers), entertainments were provided, comprising songs and dances arranged by the nurses, assisted by a conjurer and a ventriloquist. Patients numbering nearly 300 were brought from other wards and were enthusiastic in their approval of the various items of the programme.

### Royal Infirmary, Bradford.

CHRISTMAS at the Royal Infirmary, Bradford, was celebrated in a manner well befitting the great anniversary. In spite of the cloud of war and the many and urgent calls which are made upon the charitably disposed, the friends of the infirmary had generously given of their best to make this day one of joy to all those who were compelled by accident or disease to spend their Christmas in the wards of the hospital, whilst each member of the staff entered enthusiastically into the spirit of the season. The wards were brightly decorated with evergreens, flowers, and coloured paper, and presented a gay and festive appearance to the patients and their friends, who were allowed to visit them in the afternoon.

THE MAYORAL VISIT.

Following a time-honoured custom, the Lord Mayor and Lady Mayoress of the city, accompanied by a large party, made a tour of the wards of the hospital and wished each patient "A very happy Christmas." The Chairman of the house committee (Mr. George Priestman) welcomed the Lord Mayor to the hospital in a speech in which he gave a brief résumé of the work which had been done during the year, which had been carried out under great difficulties owing to shortage of staff. He congratulated everyone concerned on the success which had been achieved.

A substantial Christmas dinner, which had been provided by the Lord Mayor, was greatly enjoyed, and afterwards every man in the hospital received a gift of tobacco and cigarettes. Father Christmas, in the person of the resident surgical officer, made a round of the wards in the morning, and every patient in the hospital received a useful Christmas gift. Boxing Day was "Children's Day," when the many beautiful toys, books, etc., which had decorated the Christmas-tree were distributed amongst the little patients by Father Christmas. The little ones had the time of their lives and revelled in the beautiful presents. Numerous concerts were held during the week.

### The Royal Victoria Infirmary, Newcastle-on-Tyne.

By A SOLDIER PATIENT.

NEVER, surely, in the history of our great institutions of healing, has the spirit of Christmas been honoured with such heartiness as during the third war Christmas. This applies in fullest measure to the military wards of this great Northern infirmary. War-scarred soldiers from the heat and sand of the most easterly Front, the precipitous ridges of Gallipoli, and the muddy trenches of France have found a veritable haven of refuge in a number of wards which are reserved exclusively for naval and military patients.

Everything that human art could contrive or sympathy suggest was laid under contribution to give the men a Christmas celebration that should lack nothing in heartiness and good cheer. The organisation was perfect. Nothing was left to chance, and the remotest details were carefully planned with discriminating insight.

The chaste dignity of the wards was embellished by artistic decorations, evergreen festoons, national flags, and fancy lamp-shades giving a most seasonable appearance to the scene of the festivities. The colour scheme of artificial lighting was appropriately carried out with red transparencies, so that the celebration was staged in the most literal sense "round the red lamp."

On Christmas Eve the nurses and maids of the infirmary staff, marshalled by the chaplain, went in procession through the wards, singing well-known carols. Carrying lighted Chinese lanterns through the darkened wards, they made a very pretty and effective display.

The arrangements for Christmas Day were of a most comprehensive character, and were carried out with the utmost enthusiasm by all concerned. A very generous provision of Christmas fare had been made, and was greatly appreciated by the patients. The hon. senior surgeon, Lieut.-Colonel Martin (R.A.M.C.), presided over the proceedings, and was supported by Mr. Orde (house governor), Miss Brown (matron), the ward sisters and nurses, who with unwearying energy devoted themselves to their multitudinous duties in the service of the men.

THE KING'S MESSAGE.

During the course of the meal Colonel Gowans, Officer Commanding 1st Northern General Hospital, read the gracious message from the King and Queen to the sick and wounded sailors and soldiers, as well as a moving tribute from the Premier of Queensland, which evoked hearty applause.

After dinner the company resolved itself into a great family party, animated by the pure spirit of enjoyment. Patients in paper caps taken from bon-bons made a picturesque group as they sat round the pianola singing, as only British Tommies can, songs of the Homeland.

To the great enjoyment and delight of the patients, various concert parties maintained a "continuous programme" of musical and other items until "Lights out." It is quite safe to say that in no place in the land was there greater happiness than in the military wards of this infirmary on Christmas Day. Most assuredly the men who have been fêted so royally will carry away with them a golden memory of the third war Christmas in the Royal Victoria Infirmary, Newcastle-on-Tyne.

### Norfolk and Norwich Hospital.

Christmas was heralded in the early hours of the morning by some of the nurses, who sang carols outside the wards. The Christmas atmosphere in every hospital proclaims itself on entering the building on Christmas morning. The hall porter—usually the most staid of individuals—greets all-comers, patients or officials, with "A Merry Christmas." Entering a ward one sees a remarkable change from ordinary times. Christmas is evidenced by the decorative ability of the sisters and nurses, which produced here a most effective result, although the decorations were on a smaller scale than in times of peace.

From one or other of the wards comes the sound of a merry chorus, dispelling any sad thought that may have entered one's mind unsought. Home-sickness is forgotten. Excitement is great when the post comes in, bringing with it parcels from home and friends. Delayed it may be, but the expectation keeps everyone on the tiptoe of excitement. As the morning goes, thoughts turn to the dinner-hour. What fare! By the kind thought of a generous donor turkeys sufficient for all have been provided. These are dissected in no mean manner by a member of the honorary medical staff or one of the officials. It is hard work carving for a hungry ward, but it is carried out with the greatest cheerfulness. Possibly the thought of a similar spread makes it all the more agreeable.

Everybody satisfied? Yes. Now for the pudding. Here it comes. A match is applied and some mysterious liquid bursts into flame—with a shriek of approval from a juvenile patient, and a grunt in the same strain from "Granny." The pudding devoured, a silence reigns for a little while, and then comes the popping of crackers. Caps, whistles, and other articles are produced, and the fun is fast and furious. The sound of bagpipes creates a silence in the wards, and some pipers from a regiment stationed in the city appear—marching along to the tune of their pipes, the strains of which are greatly enjoyed by the patients. The sick and wounded soldiers seem to be having the time of their lives, and in their wards the fun is uproarious, yet never exceeding the bounds of discipline, which naturally are relaxed considerably. Songs, recitations, and other forms of entertainment are carried out with all the vigour imaginable. Some gifted patient amuses everybody by his antics, and, incidentally, himself enjoys the fun to the full. A song—contrasting the funny man's antics—produces a hush which is broken at the finish by loud applause, showing very clearly the fact that practically every man loves a good song. No matter in which ward it is, the atmosphere is the same. Even the quiet of the women's wards is broken, and the change is enjoyed.

The distribution of presents from a Christmas-tree in one of the military wards creates endless fun and merriment. Father Christmas, with unlimited wit, presents patients, nurses, and officials with little gifts—some funny, some useful, and all acceptable.

No Public Appeal.

The above is a brief outline of the general appearance of this hospital at Christmas. Naturally there are a few patients who require to be kept quiet, and their interests are studied, but even these afflicted ones are made to feel the Christmas good will prevailing throughout the institution. This year no appeal was made to the general public for extra comforts or for the funds to purchase them, but, nevertheless, we were not forgotten, and, thanks to those good people who contributed, the good cheer referred to above was obtained. No doubt the public would have responded freely had they been asked, but throughout the year they have shown unstinted generosity in their support of the hospital, and as Christmas in wartime must encroach heavily on their pockets it was thought fit to refrain from a public appeal.

The staff as well as the patients have their good time, chief of which is in making others happy. In one of the departments of the hospital a splendidly arranged tea was given to the members of the staff. Music by an accomplished pianist and songs made the whole thing go with a swing. The day finishes, and all wonder why that sense of satisfaction is so strong within them. Why should they wonder? They have put themselves to the task of giving happiness to those less fortunate than themselves, and their efforts have been crowned with immeasurable success.

Peace and good have triumphed as they always will, and one looks back with real joy to those happy faces beaming all round the long wards—suffering forgotten in the happiness of the moment.

### County Hospital, Lincoln.

Christmas Day was ushered in by a party of carol singers, who visited the various wards. After this, presents were distributed to all the patients. At ten o'clock a Christmas service was held in the hospital chapel, at which every patient who was well enough attended.

Dinner consisted of turkey, plum-pudding, mince-pies, and other seasonable delicacies. The message from His Majesty the King to his wounded soldiers was read out in each of the military wards and received with great enthusiasm.

In the afternoon there was music throughout the hospital.

On Boxing Day the patients' friends were invited to tea in the wards. In the children's ward a Christmas-tree was provided, and the little ones had a right royal time in receiving presents and sharing in the general excitement.

### General Hospital, Wolverhampton.

The Yuletide celebrations at the hospital began on Christmas Eve with the singing of carols in the various wards by members of the nursing staff. This time-honoured custom was greatly appreciated by the patients.

At 7 a.m. on Christmas morning Holy Communion was celebrated in the chapel, followed at 10 a.m. by morning prayers. During the morning each individual patient in the hospital received a present of some article of practical use. In the male portion of the institution the

CHRISTMAS IN THE HOSPITALS— (*continued*).

patients, except, of course, those rendered incapable by illness, were soon busy smoking, pipes and tobacco having been previously issued.

At 12.30 joints of roast beef and plum-puddings were served out to each ward, members of the staff having undertaken the office of carving. The hearty manner in which the patients disposed of this fare amply signified their appreciation. Those of the patients who were unable to enjoy this repast were provided in all possible cases with Christmas fare of other description. During the afternoon the hospital was visited by the choir of St. Peter's Church, Wolverhampton, and carols and sacred songs were given in the wards till about 6 o'clock. Between the hours of 4 and 7 o'clock tea was served to the staff in daintily set-out "tea-rooms" adjoining each ward, the nurses visiting their colleagues in the different departments and comparing over cups of tea their efforts of decoration and the experiences of the day. The last important item took place at 7.30 p.m.—the residents' and sisters' dinner. This finished, the remaining hours of the evening were pleasurably spent in the customary Yuletide fashion.

The Decorations.

Every ward presented an equally delightful appearance. In the children's ward strings of Chinese lanterns, festoons of gaily-coloured ribbon and evergreens led from all parts to a huge Christmas-tree situated at one end. The tree, presented by the Chairman and dressed by members of the Ladies' Visiting Committee, was heavily laden with toys, and the Teddy-bear enthroned upon its summit actually touched the ceiling. Electric lamps of various colours shone brightly among the branches and greatly enhanced the general effect, which called forth the complete approval of the juvenile spectators. The soldiers' ward was a vision of bright colouring, flags of all the Allied nations, streamers of every hue, evergreens, etc., being everywhere in profusion, whilst the blue suits and red ties of the soldiers fitted in admirably with the general scheme. Among the many presents sent by thoughtful well-wishers each soldier received Christmas greetings from the Mayor and Mayoress of Wolverhampton, chocolates from Messrs. Cadbury, and a parcel of smoking material from the local "Soldiers' Comforts Fund." Although sick and wounded, the inmates of this ward clearly manifested the spirit of happiness prevailing among them.

Sunday Festivities.

Festivities were continued during the remainder of the week. On Boxing Day the chief event was the nurses' dinner, at which fancy dress was worn, prizes being given for the most original. The first prize was divided between two nurses for their excellent representation of a male and female "casualty" case respectively. The maids' and porters' dinner was held later in the week. Concerts were held in the different wards, the choir of All Saints' Church kindly assisting. A whist-drive, held in the Nurses' Home, was thoroughly enjoyed by the staff. As a fitting termination to the whole series of events came the distribution of gifts from the Christmas-tree, when every child received a present to commemorate the festival of Christmas.

Such a Christmas, of course, called for a vast amount of extra work from every member of the staff. However, enthusiasm and united effort overcame all existing difficulties, and the labour was amply requited by the resultant happiness of both the patients and the staff.

### Lord Mayor Treloar Crippled Children's Hospital, Alton, Hants.

The Christmas festivities were as bright, joyous, and altogether seasonable to both the little patients and the staff alike as capable management and enthusiastic labour could make them. They were rendered additionally interesting to one and all by reason of the fact that Sir William Treloar, the founder of the hospital, and Sir William H. Dunn, the Lord Mayor of London, one of the trustees and the treasurer of the hospital, spent their Christmas at Alton Park and took part in the festivities.

An Agile Father Christmas.

The proceedings opened in an informal but very entertaining way on the Friday before Christmas, when Sir William Treloar, in the costume of Father Christmas, walked in the hospital grounds followed by a flock of sheep and surrounded by crippled children. Sir William also drove round in a donkey-cart full of toys, and eventually climbed up a ladder to enter a ward by way of the window, to the immense delight of the children.

The wards had been decorated with evergreen, artificial flowers, and coloured lanterns, with dainty and artistic effect, and in a ward of each of the four blocks stood a wondrously laden Christmas-tree, a centre of fascinating interest to every child beholding it.

On Christmas Eve a choir of nurses went in procession carol-singing round the wards and the hospital grounds. All lights were put out in the wards as the choristers came round wearing their hooded cloaks like cowled monks and swinging coloured lanterns, and the picturesque effect lent a rare charm to their sweet carolling. On Christmas morning there was a service for the staff with a celebration of Holy Communion in the hospital chapel, and afterwards a service for the children.

Delightful Children's Entertainments.

At mid-day the children had their Christmas dinner, which was of the traditional kind, and, of course, thoroughly enjoyed. The children of each of the four blocks had been gathered into that ward in each block in which the Christmas-trees had been placed, and during the afternoon troupes of nurses representing each block gave short entertainments of the kind beloved by children —entertainments with pretty costumes, spirited singing, and lots of dancing. There were the jesters, quaintly attired, who sang and danced, and jingled their bells with such untiring energy that it seemed they must drop for want of breath long before their performance finished; there were the Quarrelsome Flowers, in a dainty little operetta, whose sprightly yet graceful dancing and delightful singing charmed everyone; there were the Sailor Girls, who in smart nautical costumes gave a selection of appropriate songs and dances, including the sailors' hornpipe, with such spirit and "go" that the audience had much ado to restrain themselves from joining in; and finally (though finally only in the sense of being mentioned last) was a troupe representing Nursery Rhymes, whose charming performance in dainty costume delighted everyone, young or old.

The Crown of the Day.

Following these entertainments came what was perhaps the event of the day—the dismantling of the Christmas-trees by Father Christmas. Sir William Treloar, who for over a quarter of a century has been a true Father Christmas to thousands of little crippled children all over the country, played this part, to the delight of everyone concerned, with all his accustomed geniality. Sir

William was ably assisted in the task of distributing the gifts by the Lord Mayor, who thoroughly entered into the spirit of the "game," and evidently enjoyed it as much as did the children. That the children enjoyed it was shown, if any evidence were needed, by the hearty and spontaneous laughter with which they greeted the humorous by-play between Father Christmas and the Lord Mayor, and the ringing cheers which followed the distribution of the gifts.

### Entertainments at the Leicester Institutions.

A CONTINUED round of entertainments has been a marked feature of the Christmas festivities. At the Base Hospital Christmas was ushered in by an excellent concert arranged by Mr. Alfred Orchin. On Christmas Day the Mayor and Mayoress paid welcome official visits to all the war hospitals. A Sunday evening concert was arranged by the Co-operative Prize Choir. Other concerts were given during the week by Mr. and Miss Russell Wray (the latter the principal girl of the Opera House pantomime, "Jack and Jill"), Madame Gwladys Nicholls, and others. Occasion was taken by the Mayor and Mayoress to present two wounded Canadian soldiers with the Military Medal for conspicuous bravery; 1,200 patients and staff were present. Lieut.-Colonel Harrison, the Administrator, is to be heartily congratulated on the excellent provision made for the enjoyment of the soldiers under his care.

At the North Evington War Hospital Father Christmas (impersonated by the night sister) visited all the wards and distributed bounties. The Christmas dinner was followed by "high tea," and by a fancy dress dinner and dance for sisters and nurses. A series of concerts was given in the dining-hall and in the wards during the week. Captain Moffat Holmes and the medical and nursing staffs were most successful in their arrangements to make the festive season a time of real joy to their patients.

At the Royal Infirmary carols were sung by the nursing staff. On Boxing Day each patient invited a friend to tea, provided by the institution. Christmas-trees in the children's and military wards were bountifully laden. A much-looked-forward-to event was Mr. Percy H. Wyke's annual concert specially arranged for wounded soldiers, which was given in the "George Oliver" ward. The stage was magnificently dressed by a choice selection of flowers lent by Mr. F. S. Brice, J.P. The artistes were Mr. Ben Davies, the celebrated tenor (who unfortunately had not recovered from the effects of a severe cold), Miss Margaret Balfour, Miss Maud Arnold ('cello), Mr. C. W. Wade, Mr. J. H. Taylor, and Professor Hamylton, the "Modern Magician." Mrs. W. N. Toller and Mr. Wykes were at the piano. Colonel Bond thanked Mr. Wykes and the artistes in a clever speech which "touched the right chord" throughout. Mr. Wykes thanked Miss Vincent, the matron, and the nursing staff for their hearty co-operation.

At the Groby Road Sanatorium the St Martin's Old Boys' Association provided many good things at the Saturday night concert and at a sacred concert on Christmas Day.

At the Gilroes Hospital Mr. W. H. Brain undertook the entertainments. Dr. Millard (M.O.H.) acknowledged the splendid efforts of the entertainers.

In addition to all these arrangements whist-drives were arranged in the respective wards of the institutions, and the "bran tub" was a popular source of enjoyment.

A MATINÉE TO 1,000 SOLDIERS.

On Friday afternoon upwards of 1,000 wounded soldiers were entertained to a matinée at the local Palace Theatre, by permission of Mr. Oswald Stoll. The entire "cast" of artistes contributed to the programme and distributed 10,000 cigarettes, provided by themselves, to the men on arrival. Colonel Harrison thanked the artistes and Mr. Trueman Towers (the local manager) and his staff for their enjoyable entertainment.

The Poor-Law institution was visited by the Mayor and Mayoress, and suitable provision was made for a thoroughly enjoyable time by the inmates.

The Free Dinner Association, as usual, looked to the comfort of the poor and deserving and provided 450 hot dinners in the homes of the recipients.

On New Year's Day Scotch soldiers were entertained at the Presbyterian Schoolroom, where an enjoyable tea and an excellent entertainment were provided under the supervision of the Rev. W. Williamson, Presbyterian chaplain.

Christmas week was indeed a memorable time for the institutions of Leicester, and will not soon be forgotten by the patients, who are generous in their praise of the town's hospitality.

